# Assessing the validity and reliability of the Turkish versions of craving beliefs and beliefs about substance use questionnaire in patients with heroin use disorder: demonstrating valid tools to assess cognition-emotion interplay

**DOI:** 10.1186/s13011-018-0166-1

**Published:** 2018-08-22

**Authors:** Melike Küçükkarapınar, Hale Yapici Eser, Vahap Ozan Kotan, Merve Yalcinay-Inan, Rifat Tarhan, Zehra Arikan

**Affiliations:** 1 Department of Psychiatry, Muş State Hospital, Muş, Turkey; 2 Department of Psychiatry, Gazi University, Faculty of Medicine, Ankara, Turkey; 3 Koç University School of Medicine, İstanbul, Turkey & Koç University Research Center for Translational Medicine (KUTTAM), İstanbul, Turkey; 4 Department of Psychiatry, Ankara Numune Training and Research Hospital, Ankara, Turkey; 5 Department of Psychiatry, Başkent University, School of Medicine, Ankara, Turkey; 6 Safranbolu State Hospital, Kastamonu, Turkey; 7 Department of Psychiatry, Koç University Hospital, İstanbul, Turkey

**Keywords:** Beliefs about substance use, Craving beliefs questionnaire, Heroin, Opioid use disorder

## Abstract

**Background:**

Cognitions associated with craving and substance use are important contributors for the psychological theories of Substance use disorders (SUD), as they may affect the course and treatment. In this study, we aimed to validate Turkish version of two major scales ‘Beliefs About Substance Use’(BSU) and ‘Craving Beliefs Questionnaire’(CBQ) in patients with heroin use disorder and define the interaction of these beliefs with patient profile, depression and anxiety symptoms, with an aim to use these thoughts as targets for treatment.

**Methods:**

One hundred seventy-six inpatients diagnosed with heroin use disorder and 120 participants in the healthy comparison group were evaluated with CBQ, BSU, Beck Anxiety Inventory (BAI), Beck Depression Inventory (BDI) and sociodemographic data questionnaire. Patient group was also evaluated with Addiction Profile Index. Reliability and validity analysis for scales were conducted. Linear regression analysis was conducted to evaluate the determinants of BSU and CBQ scores.

**Results:**

Cronbach alpha level was 0.93 for BSU and 0.94 for CBQ. Patient group showed significantly higher CBQ, BSU, BAI and BDI scores (*p* < 0.001). BSU score significantly correlated with API-substance use profile score, API-diagnosis, BAI, BDI and CBQ (*p* < 0.005), whereas CBQ scores significantly correlated with API-diagnosis, API-impact on life, API-craving, API-total score, BSU, BAI, BDI and amount of cigarette smoking (*p* < 0.002). Number of previous treatments and age of onset for substance use were not correlated with either BSU or CBQ. BAI and BDI scores significantly predicted BSU score, however only BDI score predicted CBQ score (*p* < 0.003).

**Conclusions:**

Craving beliefs were highly correlated with addiction profile. Anxiety and depression are significant modulators for patients’ beliefs about substance use and depression is a modulator for craving and maladaptive beliefs, validating emotion-cognition interplay in addiction.

## Background

Substance use disorders (SUD) are major public health problems and a significant cost both for the individual and the society [1]. Lifetime prevalence of substance use is around 2.8% in Turkey. Of the patients who are admitted for treatment for the first time, 73% are reported to be diagnosed with heroin use disorder and heroin is the substance found to be most related with more than half of drug induced deaths in young adults [2, 3] .One of the latest reports point out that the prevalance of high risk opioid use is in Turkey is around 0.03% and it is the least reported rate among European countries, however since 2009 both drug use and high risk opioid use have dramatically increased in Turkey [3, 4].

Substance use disorders are results of a process, where multiple factors take part both in the initiation and also in the maintenance. With repeated use, substance use turns into a chronic disorder, which is reinforced by both biological and psychological factors. Currently pharmacotherapies are used to relieve the withdrawal symptoms and also to decrease craving in the maintenance phase, with limited efficacy [5, 6]. As a pharmacological approach, opioid substitution treatment using buprenorphine-based medication has been used in Turkey since 2010 Alcohol and Drug Research, Treatment and Training Centre (AMATEM) clinics [3]. On the other hand, cognitive and behavioral approaches are also effective in the management of opioid use disorders, by increasing the duration of opioid abstinence days [7]. Current evidence shows that pharmacological treatment is more effective when it is used in combination with psychological treatments than pharmacological or psychological treatments alone, particularly for opiate users [8]. However, higher number of studies that assess the effectiveness of either cognitive behavioral therapy (CBT), the core beliefs and determinants related with substance use are needed.

CBT of substance use disorders was suggested by Aaron Beck et al., who had developed a theory about the psychological origins of SUD and the interaction of cognition or thought, emotion and behavior for this disorder [9]. It is generally accepted that substance use turns into a vicious cycle in patients diagnosed with SUD. Even though withdrawal, craving or compulsive use may decrease with pharmacotherapy, patients suffer from their occupation with automatic thoughts, which are easily triggered by conditional stimuli, emotional states or any frustrating life event. Even experiencing these automatic thoughts about SUD can be very stressful for the individual and they may be overvalued as a sign that they will not succeed in treatment of SUD. CBT of SUD involves avoiding the high risk situations and triggers of use, dealing with urge and craving, focusing on automatic thoughts as beliefs about substance and beliefs about craving [9, 10] and replacing them with more adaptive alternative thoughts. Beck and Mitcheson suggest that focusing on beliefs about substance is the core to avoid the relapse [9, 11]. From the perspective of cognitive behavioral therapy, these automatic thoughts are also named as maladaptive thoughts since they are inflexible, negatively biased, and negatively affecting the course of the disorder [12] These beliefs may also influence not only the patients’ but also the treatment professionals and general public’s view about addiction [13].

Based on this observation, Beck et al. generated two questionnaires: Beliefs about substance use (BSU) to define substance addict’s beliefs about substance use and rate beliefs statements and Craving Beliefs Questionnaire (CBQ) to define the role of craving related automatic thoughts. Both scales are Likert type and self-report [14]. Originals of these questionnaires can be found in A. Beck’s “Cognitive Therapy of Substance Use” book [9]. Aslan S. et al [15] previously translated these scales to Turkish for its use in patients diagnosed with alcohol use disorder and it was found to be reliable.

Previous literature about craving beliefs suggests that craving beliefs modify the course of methamphetamine use [16]. But it is not known if craving beliefs affect the course only in less dependent individuals as seen in smokers [17] or if it is effective for people with severe SUD as well. In addition, depressive disorders and anxiety disorders are known to lead to negative automatic thoughts and decreased self confidence for problem solving [18] however how these symptoms interact with craving beliefs and beliefs about substance use is also not studied as far as we know.

Opioids have an anxiolytic effect at the beginning, but chronic opioid use causes depressive symptoms and chronic anxiety. Moreover; negative mood, high level distress intolerance are also associated with low treatment incidence and increased relapses in substance abusers [19, 20] Experiencing an anhedonic state may play a role in the onset of addiction and reduce the capacity of individuals to overcome the symptoms of early stages of abstinence [21]. Heroin related cognitions have important roles in the frequency of heroin use [22]. For this reason, it is necessary to detect affective symptoms and cognitive distortions that may affect addictive behavior in the assessment of the patients with SUD.

To our knowledge, BSU und CBQ are the only scales that assess distorted thoughts specific to substance use. These scales separate thoughts related with maintenance of substance use into two categories and they give researchers and clinicians a chance to find moderators of different clinical courses.

Based on the aforementioned gap in the current literature and as clinicians working with patients diagnosed with SUD and using CBT methods in their follow-up interviews, we felt the need to reestablish the Turkish validity of the questionnaire for opioid use disorders. With this aim, we tested the validity and the reliability of Turkish version of BSU and CBQ in patients diagnosed with heroin use disorder by comparing this group with healthy individuals. As a second aim, we aimed to define the effect of beliefs about substance use and craving beliefs on the course of opioid addiction and to define the modulatory role of depressive and anxious symptoms on beliefs about substance use and craving beliefs, in order to address the emotion-cognition interplay in SUD.

## Method

### Translation steps

Authors received permission from Guilford Press, the original right holder of the scales, for the scales translation [23]. BSU and CBQ were translated to Turkish by two authors. The translated scales were compared to previously used Turkish scales in patients with alcohol use disorder [15]. One author translated scales back to English, and then a professional translator compared them with the original versions. Inconsistencies were corrected by consensus.

### Participants

The study was performed at Alcohol and Drug Research, Treatment and Training Centre (AMATEM) of Ankara Numune Training and Research Hospital and Gazi University Faculty of Medicine Department of Psychiatry between September 2015 and February 2016. We tried to recruit at least 5 subjects per item of the scales as suggested by Gorsuch [24] and recruited all inpatients that provided a written informed consent during the study period. All participants were older than 18 years of age. One hundred seventy-six non-alcoholic inpatients diagnosed with heroin use disorder according to DSM-5 [25] criteria by a psychiatrist and 120 healthy volunteers who had never used alcohol or any other substances, and who did not have a psychiatric diagnosis, were included in this study. Healthy comparison group has been selected from hospital workers, relatives of the psychiatric and other medical disorder outpatients and volunteers who applied after advertising the study. Patients with a comorbid diagnosis of mental retardation, schizophrenia, bipolar disorder or dementia were excluded. Study groups were matched for age, gender and income level. The local ethics committee of Gazi University Faculty of Medicine has approved this study by IRB date and number: 09.05.2013/01.

### Procedures and measurements

Patients and healthy comparison group were informed about the purpose and procedures of the study and an informed consent was obtained from every participant. A sociodemographic data form to note the general properties of the sample was used to assess all patients and healthy comparison group. For the patient group, all scales were given to the patients at the second week of treatment after completion of detoxification. It was reassured that the patients were not in a withdrawal state or actively craving for the substance. The following scales were administered to both groups for reliability and validity assessment and also to define the relationship of depressive and anxious symptoms with beliefs about substance use and craving beliefs.

*Socio demographic data form* was generated by the authors to obtain general demographical data such as age, gender, educational status, marital status, and occupation and substance use disorder related variables. A psychiatrist filled each form during the face-to-face interview with participants. The questionnaire also included questions to record the substance use profile of the patients. For the patient group, age of substance use onset and duration since the onset of first substance use, names and durations of the used substances, the reason of using substance, number of previous applications for treatment, presence of physical and mental illness due to the substance use, forensic events related to substance use, substance use in family and neighborhood were also questioned with a self prepared survey.

#### Craving beliefs questionnaire

CBQ is also a self-report questionnaire, where patients rate their agreement with each 20 item on a 7 point Likert scale. This questionnaire measures beliefs about substance cravings [6, 10]. Higher scores indicate feeling more helpless to deal with craving.

#### Beliefs about substance use questionnaire

The BSU is a self-report questionnaire, where patients rate their agreement with each statement [14]. Turkish version of the scale translated for patients with alcohol use disorder, has shown to have good internal consistency (Cronbach’s alpha 0.86) and reliability [15]. This 20 item, 7 point Likert scale involves items about decreased self-efficacy and self-confidence to stop using substance or to cheer up the life, in addition to self-blaming thoughts.

#### Beck anxiety inventory (BAI) and Beck depression inventory (BDI)

Symptoms of anxiety and depression were assessed by BAI [26] and BDI [27], respectively. Both of these scales consist of 21 multiple-choice questions and they are accepted as reliable and valid tools to assess anxious and depressive symptoms. The Turkish version of both scales were developed and psychometric properties were studied [28].

### Addiction profile index (API)

In order to obtain more data about substance use disorder profile of the patients and to evaluate the correlation of BSU and CBQ, API has also been applied to SUD patient group. This scale has been developed by K. Ögel [29]. It is a self-report questionnaire consisting of 37 items and measuring a total score for addiction and 5 subscales composed of (1) characteristics of substance use; (2) diagnostic criteria of substance use disorder; (3) the effects of substance use on the user; (4) craving and (5) motivation to quit using substances. In the first subscale, the number and frequency of used alcohol/ substances and the problems created by them are evaluated. Second subscale contains the diagnostic criteria of substance use disorder according to DSM and ICD. Third subscale includes questions that determine the severity of addiction problems such as education, work, family, economic, legal problems, pattern of substance use and criticism from the family or the environment. With the fourth subscale, it assesses craving of substances. The last subscale evaluates the motivation to stop the substance use. Thus, API allows measuring the severity of different dimensions of substance use disorder.

### Data analysis

SPSS statistical program package licensed to Koç University has been used for all analysis. Groups were compared for means of continuous variables using t-test and for distribution of non-continuous variables using chi-square or Fisher’s exact test. Factor analysis of CBQ and BSU has been analyzed using Bartlett’s sphericity test and Kaiser-Meyer-Olkin (KMO) test for sampling adequacy. *p* < 0.05 was chosen as the significance level for Bartlett’s sphericity test [30] and KMO scores between 0 and 1 and factor analysis level of 0.6 was chosen significant. According to Tabachnick and Fiddell, threshold is .32 to decide type of rotation [31]. The Principal Component Factor Analysis with Promax Rotation was conducted to examine factorial structure of BSU and CBQ. Cronbach alpha coefficient was calculated for internal validity of BSU and CBQ.

Patient and healthy groups were compared for the mean of continuous variables by t-test. Pearson correlation analysis was conducted for the analysis of correlation of BSU and CBQ scores. To test our second aim, only patient group was analyzed separately for the analysis of correlation of BSU or CBQ scores with age, age of substance use onset, duration of substance use, anxiety scores, depressive scores, API- total and subscale scores and total cigarette smoking amount. A multivariate linear regression analysis has been conducted to define the predictors of BSU and CBQ scores. For the analysis where smoking amount and age of onset for substance use data were used, data of 169 heroin SUD patients could be used due to missing data. *p* < 0.01 was considered significant due to multiple comparisons.

## Results

### Sociodemographic variables table for age, gender, educational level, income level

There were no significant differences between patients and comparison group regarding mean age (23.8 ± 4.4 and 24.8 ± 4.7, respectively, *p* = 0.06, df: 294, t-test), gender distribution (Male/female distribution: 169/7 and 118/2, *p* = 0.32, Fischer’s exact test), income level (*p* = 0.17, Fischer’s exact test) and marital status (*p* = 0.2, Fischer’s exact test). Educational level of the patients was significantly lower than comparison group (*p* = 0.001, Fischer’s exact test).

### Validity for CBQ

**Fig. 1:**
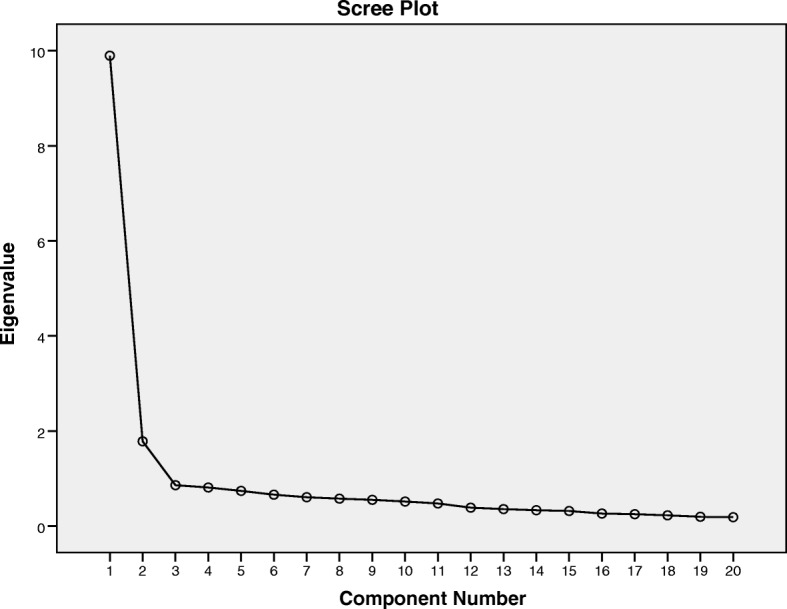
Scree plot for CBQ

We conducted factor analysis to examine the scale characteristics of the data. Factor analysis showed that the Kaiser-Meyer-Olkin measure of sampling adequacy was .94 and that Bartlett’s test of Sphericity [32], which assesses if the dataset is suitable for factor analysis, was significant (*p* < 0.0001). As these tests were significant, we interpreted the factor analysis results. Principal components analysis with promax rotation conducted for examining factor structure of CBQ with 20 items. It extracted two factors with eigenvalues over 1 and explained 58.4% of total variance (Table 1). Also, inspection of the screen-plot indicated two factors (Fig. 1). For CBQ, we run 2 factors PCA followed by a promax rotation again. The resulting correlation matrix for the factors shows that there are .66 correlations between factor 1 and 2.

**Table 1:** Items and Factor Loadings of CBQ

Item	Factor loading
Factor 1: Psychophysical reactions to craving	
CBQ10 Craving is my punishment for using prescription opioids	0.90
CBQ12 The images/ thoughts I have while craving prescription opioids are out of my control	0.88
CBQ2 If I don’t stop the cravings for prescription opioids, they will get worse	0.85
CBQ16 When I’m really craving prescription, I can not work.	0.84
CBQ13 The craving for prescription opioids make me so nervous I can’t stand it	0.82
CBQ11 If people have never used prescription opioids then they have no idea what craving really is.	0.81
CBQ4 The craving makes me use prescription opioids.	0.75
CBQ9 I can’t stand the physical symptoms I have while craving prescription opioids.	0.71
CBQ7 Once the craving for prescription opioids start, I have no control over my behavior.	0.65
CBQ3 Craving for prescription opioids can drive you crazy	0.64
CBQ1 Craving is a pyhsical reaction, I can not do anything about it	0.55
CBQ17 Either I’m craving prescription opioids or I ‘m not; there is nothing in between.	0.53
Factor 2: Weakness to cope with craving	
CBQ15 Since I’ll have the craving the rest of my life I might as well go ahead and use prescription opioids	0.89
CBQ8 I’ll have cravings for prescription opioids the rest of my life	0.83
CBQ14 I’ll never be prepared to handle the craving for prescription opioids	0.77
CBQ19 When craving prescription opioids, it’s OK to use prescription opioids to use.	0.72
CBQ6 I don’t have any control over the craving for prescription opioids	0.70
CBQ20 The craving for prescription opioids are stronger than any my will power	0.63
CBQ5 I’ll always have cravings for prescription opioids	0.57
CBQ18 If the craving gets too intense, using prescription opioids is the only way to cope with the feeling.	0.46
Variance	58.3%

As the assessment of discriminative validity, mean CBQ scores of the healthy group (22.7±7.5) were significantly lower than patients with substance use disorder (64.3±25.5, *p* < 0.001).

### Reliability for CBQ

Internal consistency: In order to determine the instrument’s internal consistency reliability, the Cronbach alpha coefficient was calculated. Its reliability was .94. The internal consistency of CBQ subscales was .93 for Subscale 1: Psychophysical reactions to craving, .88 for Subscale 2: Weakness to cope with craving.

For subscale 1, intraclass correlation coefficient (ICC) was used to determine the inter-rater reliability coefficient. It showed that there are strong reliability based on the 95% confident interval of the ICC estimate (ICC = 0.934, *N* = 295, *P* < 0.001). For subscale 2, intraclass correlation coefficient (ICC) was used to determine the inter-rater reliability coefficient. It showed there are strong reliability based on the 95% confident interval of the ICC estimate (ICC = 0.875, *N* = 295, *P* < 0.001).

Item discrimination: Item-total correlation is accepted as a primary criterion and must be equal to 0.30 or at least greater than 0.25. For CBQ, the item discrimination indices ranged from .49 to .80. The corrected item-total correlations have shown that each of the items of CBQ revealed good reliability (Table 2).

**Table 2:** Item-total correlation and Cronbach’s alpha if item deleted for CBQ

CBQ item	Item-total correlation	Cronbach’s Alpha if item deleted
CBQ1	0.59	0.94
CBQ2	0.77	0.93
CBQ3	0.73	0.93
CBQ4	0.80	0.93
CBQ5	0.62	0.94
CBQ6	0.58	0.94
CBQ7	0.72	0.94
CBQ8	0.61	0.94
CBQ9	0.56	0.94
CBQ10	0.69	0.94
CBQ11	0.64	0.94
CBQ12	0.78	0.94
CBQ13	0.79	0.94
CBQ14	0.59	0.94
CBQ15	0.49	0.94
CBQ16	0.75	0.94
CBQ17	0.69	0.94
CBQ18	0.60	0.94
CBQ19	0.48	0.94
CBQ20	0.68	0.94

### Validity for BSU

**Fig. 2:**
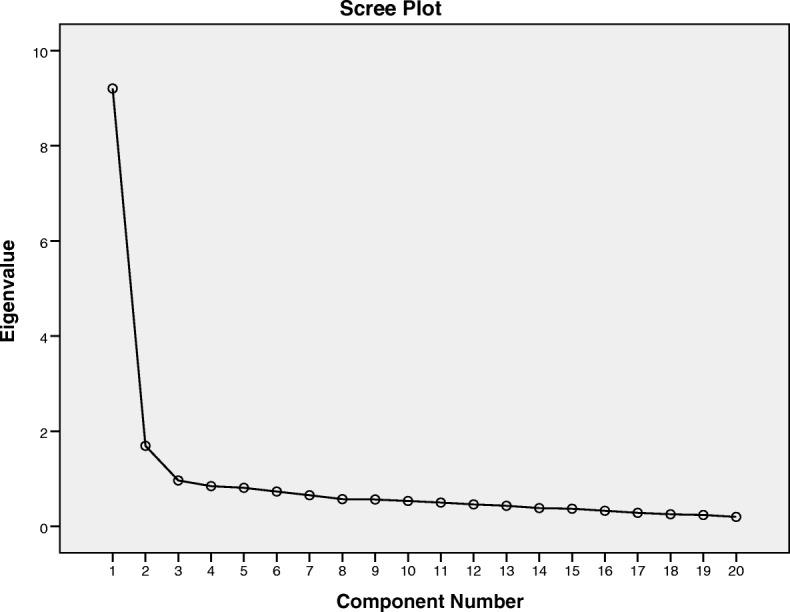
Scree plot for BSU

Factor analysis showed that the Kaiser-Meyer-Olkin measure of sampling adequacy was .94 and Bartlett’s test of Sphericity [32] was significant (*p* < 0.0001). Principal components analysis with promax rotation conducted for examining factor structure of BSU with 20 items. It extracted two factors with eigenvalues over 1 and explained 54.5% of total variance (Table 3). For BSU, we run 2 factors PCA followed by a promax rotation. The resulting correlation matrix for the factors shows that there are .64 correlations between factor 1 and 2. Also, inspection of the screen-plot indicated two factors (Fig. 2). For BSU, we run 2 factors PCA followed by a promax rotation. The resulting correlation matrix for the factors shows that there are .64 correlations between factor 1 and 2. As the assessment of discriminative validity, we found a significant difference among research groups based on t-test. BSU mean scores of heroin SUD patients (54.05±24.5) were found to be significantly higher than healthy comparison group (21.6±4.6, *p*<0.001).

**Table 3:** Items and Factor Loadings of BSU

Item	Factor loading
Factor 1: Facilitative beliefs about heroin use	
BSU10 I don’t deserve to recover from use/drink	0.91
BSU11 I’m not a strong enough person to stop	0.84
BSU13 Drug use and drinking are not problems for me	0.84
BSU16 If someone has a problem with drugs/drink, it’s all genetic	0.79
BSU9 Life would be depressing, if I stopped	0.78
BSU7 My life won’t get any better, even if stop using drugs/drinking	0.69
BSU4 This is the only way to cope with pain in my life	0.69
BSU2 Using drugs is the only way to increase my creativity and productivity	0.69
BSU12 I could not be social without using drugs/drinking	0.59
BSU17 I can’t relax without drugs/drinking.	0.39
Factor 2: Psychophysical beliefs about heroin use	
BSU19 I can’t control my anxiety without using drugs/drinking.	0.89
BSU14 The cravings/ urges won’t go away unless I use drugs/drinking	0.80
BSU15 My drug use/drinking is caused by someone else (e.g. spouse, family, etc.	0.77
BSU6 The craving/urges make me use drugs/drinking	0.66
BSU20 I can’t make my life fun unless I use drugs/drinking	0.62
BSU1 Life without using drugs/drinking is boring.	0.61
BSU8 The only way to deal with my anger is by using drugs/drinking	0.57
BSU18 Having this drug/drink problem means I am fundamentally a bad person	0.56
BSU3 I can’t function without it.	0.47
BSU5 I am not ready to stop using drugs/drinking.	0.40
Variance	54.5%

### Reliability for BSU

#### Internal consistency

Internal consistency: In order to determine the instrument’s internal consistency reliability, the Cronbach alpha coefficient was calculated. Its reliability was .93. The internal consistency of BSU subscales was .91 for subscale 1: facilitative beliefs about heroin use, .88 for subscale 2: psychophysical beliefs about heroin use.For subscale 1, intraclass correlation coefficient (ICC) was used to determine the inter-rater reliability coefficient. It showed that there is strong reliability based on the 95% confident interval of the ICC estimate (ICC = 0.909, *N* = 295, *P* <0.001). For subscale 2, intraclass correlation coefficient (ICC) was used to determine the inter-rater reliability coefficient. It showed there is strong reliability based on the 95% confident interval of the ICC estimate (ICC = 0.867, *N* = 295, *P* <0.001). Item discrimination: For BSU, the item discrimination indices ranged from .37 to .77. The corrected item-total correlations have shown that each of the items of BSU revealed good reliability (Table 4). The assessment of similar test validity has shown a positive correlation between CBQ–BSU scores (pc: 0.59, *p* < 0.001). No correlations were found between total and subscale scores of API and total scores of BSU (pc: 0.12, *p* = 0.1).

**Table 4:** Item-total correlation and Cronbach’s alpha if item deleted

BSU	Item-total correlation	Cronbach’s Alpha if item deleted
BSU1	0.62	0.93
BSU 2	0.76	0.93
BSU 3	0.73	0.93
BSU 4	0.77	0.93
BSU 5	0.62	0.93
BSU 6	0.56	0.93
BSU 7	0.69	0.93
BSU 8	0.62	0.93
BSU 9	0.73	0.93
BSU 10	0.58	0.93
BSU 11	0.64	0.93
BSU 12	0.53	0.93
BSU 13	0.62	0.93
BSU 14	0.53	0.93
BSU 15	0.36	0.94
BSU 16	0.65	0.93
BSU 17	0.67	0.93
BSU 18	0.54	0.93
BSU 19	0.62	0.93
BSU 20	0.73	0.93

### Interplay of substance related cognitions with mood, anxiety and other triggering factors

Patient group showed significantly higher CBQ, BSU, BAI and BDI scores, compared to healthy comparison group (*p* < 0.001, t-test). Based on the pearson correlation test, both CBQ and BSU scores significantly correlated with BAI scores (Pc:0.27, *p* < 0.001 and Pc: 0.38, *p* < 0.001, respectively) and BDI scores (Pc:0.31, *p* < 0.001 and Pc: 0.43, p < 0.001, respectively). CBQ and BSU scores correlated with API total scores (Pc: 0.24, *p* = 0.001 and Pc: 0.24, *p* = 0.001, respectively) and API craving scores (Pc:0.25, *p* = 0.001 and Pc: 0.26, *p* < 0.001, respectively).

### Correlation of BSU and CBQ scores with addiction profile

Based on the pearson correlation test, BSU score significantly correlated with API-substance use profile score (Pc: 0.29, p < 0.001), API-diagnosis (Pc: 0.21, *p* < 0.001) and CBQ (Pc: 0.23, *p* = 0.002). API-substance use profile score corresponds to higher number and frequency of different substances. API-diagnosis score corresponds to higher number of problems related to substance use.

CBQ scores significantly correlated with API-impact on life (Pc: 0.29, *p* < 0.001), API craving (Pc: 0.50, *p* < 0.0001), API- total score (Pc: 0.25, *p* = 0.001), BSU (Pc: 0.23, *p* = 0.002), and negatively correlated with the amount of cigarette smoking (Pc: − 0.48, *p* < 0.001). API-impact on life mainly corresponds to social problems and dysfunctions caused by substances.

Number of previous treatment attempts to stop using substances, age of substance use onset and duration of substance use were not correlated either BSU or CBQ (*p* > 0.1).

Regardless of the substance they used in addition to heroin, patients with any substance use in the last month significantly reported higher CBQ scores, (77.4 ± 20 vs 53 ± 25 respectively, *p* < 0.001), but no difference in BSU scores (51 ± 25 vs 58 ± 24.5 respectively, *p* = 0.08). Patients reporting heroin as the substance they used for the longest time, had lower mean CBQ scores compared to others (60.5 ± 26.5 vs 70 ± 23.4 respectively, *p* = 0.016).

A multivariate linear regression model was fit with covariates: age, gender, educational status, income level, amount of smoking, beck depression and beck anxiety scale scores to test if they could predict CBQ or BSU scores. Both models showed a significant effect of the variables (r2: 0.34, *p* < 0.001 for CBQ and r2: 0.27, *p* < 0.001 for BSU), however sociodemographic variables had an insignificant effect, whereas CBQ scores were significantly predicted by the amount of smoking (B coefficient: − 0.43, p < 0.001) and beck depression scores (B coefficient:0.25, *p* = 0.006). BSU scores were predicted by both the amount of smoking (B coefficient:0.20, *p* = 0.001), beck anxiety scores (B coefficient:0.25, *p* < 0.001) and beck depression scores (B coefficient:0.30, *p* < 0.001).

## Discussion

The findings in the present study indicate that the Turkish versions of BSU and CBQ are useful tools to evaluate the beliefs of patients with heroin use disorder about heroin use and cravings. Both scales showed significant discriminative validity and internal consistency reliability. These findings support that for all subgroup analysis, both BSU and CBQ are able to distinguish patients with heroin use disorder significantly from healthy comparison group. The assessment of similar test validity has shown a positive correlation between CBQ–BSU scores.

The analysis of CBQ showed that craving beliefs for drugs are grouped around two factors. First factor, which is named as ‘Psychophysical reactions to Craving’ in this study, mainly seems related with the effects of craving on daily life, mental health and physical health. Second factor, which is named in this study as ‘Weakness to cope with craving’ in this study, mainly involves negative thoughts about self-resources to cope with craving. According to our findings of BSU, these maladaptive beliefs could also be examined in two main factors. First factor, which is named in this study as ‘facilitative beliefs about heroin use’, mainly, involves items about self-worthlessness, lack of insight for substance use problem and negative thoughts about self-resources to cope with the substance. Second factor, which is named in this study as ‘psychophysical beliefs about heroin use’, involves items about the role of substance in novelty creating in life, in addition to lack of insight for substance use problem. In current study, higher frequency and variety of substance use are also positively related with these beliefs in the analysis of API substance use dimension. These subscales of both scales could be used in further studies for understanding their relationship with treatment adherence, number of relapses and duration of abstinence. Also they could be used as distinct dimensions to focus on therapy sessions for increasing meta-cognitive awareness and challenging maladaptive thoughts as suggested in trans diagnostic CBT for SUD and depression [32].

Our data suggests that the more patients with heroin use disorder had craving beliefs, the more they had depressive symptoms, anxiety and social problems in daily life. It supports the study that emphasized the quality of life was worse among patients with heroin use disorder who had psychiatric comorbidities such as anxiety or depression [33]. The risk of comorbidity of substance use disorders with depressive episodes are reported as almost 3 fold increased and it is also associated with a worse outcome for both disorders [32]. Maladaptive and distorted cognitions could be the modulator for both SUD and depressive episodes.

Depressed individuals report more negative beliefs and less metacognitive awareness [34]. Affective states also are significant modulators for decision-making processes of substance use disorders and this relationship is also bound to the duration of substance use [35]. Patients with a substance use may also present with severe impulse control disorders that are objectively measured by tasks as Iowa gambling test [36]. Our study points out that it is insufficient to focus only on substance use in addiction treatment, because craving for heroin may significantly be associated with impaired decision making, having negative mood/anxiety states and impulsive behaviors which may need a holistic approach in treatment. These thought patterns were shown to explain some of the relationship between depression and coping behaviors against alcohol use for adolescents [37] in accordance with our findings. This finding is often stated for patients with alcohol use disorder [38], however its study in patients with heroin use disorder is limited. Anxiety and depression are significant modulators for patients’ beliefs about substance use and depression is a modulator for craving, validating emotion-cognition interplay in addiction.

Another significant finding of this study was the relationship between tobacco use and heroin craving. CBQ scores were lower in patients with heroin use disorder who reported higher amounts of smoking. And there was a moderate correlation among these variables. Smoking may be a gate-away pathway to decrease heroin use, however it is also argued in literature that concomitant tobacco and heroin use seems to increase craving [39]. Tobacco smoking is often ignored in substance dependence treatment, and further attention and investigation on their association is needed.

It may be argued here that BDI is not an adequate psychometric tool to detect depressive symptoms in patients with heroin use disorder and that BDI scores in this study may not be the correspondence of a depressive episode, however BDI is a valid tool for assessment in this group [40]. The positive correlation between BAI-BDI and BSU-CBQ scores could reveal that the belief about providing a positive relief after substance use has a positive relationship with anxiety level and higher depressive automatic thoughts.

Lastly, this study has some limitations. Depressive symptoms, anxiety symptoms, craving and maladaptive beliefs were assessed through self-reports. The biological verifications would provide effectively assessment of the vicious cycle in heroin addiction. The study was cross-sectional and it does not allow us to assess the causality and relationship between depressive symptoms, anxiety symptoms, craving and substance related beliefs. Also, participants have not been tested for test retest reliability. Future study could assess participants at multiple time points to detect the changes in substance use, mental health, craving and substance related beliefs. The design of the study does not allow whether or not gender differences and treatment options affect craving and substance related beliefs in heroin addiction. Effects of personal traits and other mental health related issues could be assessed in future studies.

## Conclusion

In conclusion, Turkish version of BSU and CBQ are useful tools to detect maladaptive beliefs in patients with heroin use disorder. Subscales of BSU and CBQ should be taken into account in future studies. Depressive symptoms and anxiety are more prevalent in patients with heroin use disorder than comparison group and they correlate with distorted beliefs that maintain substance use. Due to this relationship, depressive symptoms and anxiety should be also the targets in treatment of patients with heroin use disorder. Patients’ maladaptive beliefs affect the course of illness and smoking may modulate these beliefs. These findings validate emotion-cognition interplay in addiction. Duration of substance use disorders is less effective compared to maladaptive beliefs on the severity and course of the disorder. Future studies targeting maladaptive thoughts specifically should be conducted.

## Abbreviations

API: Addiction Profile Index
BAI: Beck Anxiety Inventory
BDI: Beck Depression Inventory
BSU: Beliefs About Substance Use
CBQ: Craving Beliefs Questionnaire
DSM: Diagnostic and Statistical Manual of Mental Disorders
ICD: International Statistical Classification of Diseases
PC: Pearson correlation coefficient
SUD: Substance Use Disorder
